# Pedestrian collective motion in competitive room evacuation

**DOI:** 10.1038/s41598-017-11197-x

**Published:** 2017-09-07

**Authors:** A. Garcimartín, J. M. Pastor, C. Martín-Gómez, D. Parisi, I. Zuriguel

**Affiliations:** 1 Departamento de Física y Matemática Aplicada, Facultad de Ciencias, Universidad de Navarra, 31080 Pamplona, Spain; 2 Focke Meler Gluing Solutions S.A., Pol. Los Agustinos c/G nave D-43, 31160 Orkoien, Navarra Spain; 3 Departamento de Construcción, Instalaciones y Estructuras, Escuela Técnica Superior de Arquitectura, Universidad de Navarra, 31080 Pamplona, Spain; 4 Instituto Tecnológico de Buenos Aires, Laverdén 389, (C1437FBG) C. A. de Buenos Aires, Buenos Aires, Argentina; 5 Consejo Nacional de Investigaciones Científicas y Técnicas (CONICET), Buenos Aires, Argentina

## Abstract

When a sizable number of people evacuate a room, if the door is not large enough, an accumulation of pedestrians in front of the exit may take place. This is the cause of emerging collective phenomena where the density is believed to be the key variable determining the pedestrian dynamics. Here, we show that when sustained contact among the individuals exists, density is not enough to describe the evacuation, and propose that at least another variable –such as the kinetic stress– is required. We recorded evacuation drills with different degrees of competitiveness where the individuals are allowed to moderately push each other in their way out. We obtain the density, velocity and kinetic stress fields over time, showing that competitiveness strongly affects them and evidencing patterns which have been never observed in previous (low pressure) evacuation experiments. For the highest competitiveness scenario, we detect the development of sudden collective motions. These movements are related to a notable increase of the kinetic stress and a reduction of the velocity towards the door, but do not depend on the density.

## Introduction

Understanding pedestrian dynamics in situations where swarms gather –such as music concerts, building evacuation, transport hubs, or commercial centres– becomes essential to improve the performance of facilities and reduce the risk of disasters^1, 2^. Among the diverse existing scenarios, two different conditions can be set apart: those in which physical contact between the individuals is weak and sporadic, and those with sustained physical contact and high stress among the individuals caused by competitiveness.

In the last decade, the former situation has been the focus of most of the existing controlled experiments^3–7^ which have hitherto motivated a substantial number of numerical and theoretical works^8–11^. Despite obvious concern (asphyxia due to lung compression is a major cause of death in crowd disasters^12^) investigations where sustained contact stress among pedestrians occurs are scarce. In general, our knowledge of these situations is mostly extracted from empirical, uncontrolled observations^2, 13^ in which a transition has been demonstrated from a laminar flow to a turbulent like motion when the density increases above values around 7 persons/m^2^. Indeed, the fluctuations of pedestrian velocity, as measured by the kinetic stress, have been shown to abruptly increase with density in both, measurements of real crowds^2^ and simulations^14, 15^. However, despite inspiring numerical results^16, 17^ and the growing interest in high density and high-stress cases^12, 18^, controlled experiments of pedestrian flow through bottlenecks where individuals do exert pressure on each other have not been reported until very recently^19–21^.

In this work, we analyse the pedestrian dynamics in room evacuation drills where people were allowed to push each other in their way out^19–21^. In the literature a number of room evacuation experiments^4–6^ have led to an important advance in the understanding of this process in situations where competitiveness is low. We know, for example, that the flow rate smoothly increases with the door size^5, 6, 22^ instead of in a steplike manner, as was believed for years. Recently, the temporal dependence of the flow^22^ has been thoroughly analysed, evidencing the difficulty of finding a steady state. Also, density and velocity measurements in front of a door have helped to obtain the fundamental diagram^3^ for this bottleneck flow. Moreover, our previous works, where different degree of contact among the pedestrians was permitted, allowed to experimentally evidence Faster is Slower (FIS) effect in humans^19–21^: the evacuation slowed down when competitiveness increased. As previously predicted^1^, FIS effect was related to long lasting interruptions of the flow caused by frictional forces among the individuals. More interestingly from a fundamental point of view was the fact that FIS effect was also evidenced in other frictional systems like sheep or granular media^20^.

Despite all this encouraging research in room evacuation, there is a lack of knowledge on the way in which pressure affects the dynamics of the pedestrians in front of the bottleneck. In order to fill this gap, we have performed three series of evacuation drills with different degree of competitiveness (see Methods) and carried out a precise tracking of all the pedestrian trajectories. From these trajectories, we could get the temporal evolution of density, velocity, and “pedestrian pressure”. The latter, analogous to the kinetic component of the stress in granular materials^23^, was related to the risk of falling and proposed as a hallmark of crowd turbulence^2, 14–16^. With our work we show that in high density conditions, depending on the competitiveness, different values of this variable can be obtained for the same density and velocity values, implying that an extra parameter (apart from the density) is necessary to characterize the crowd dynamics.

**Figure 1:**
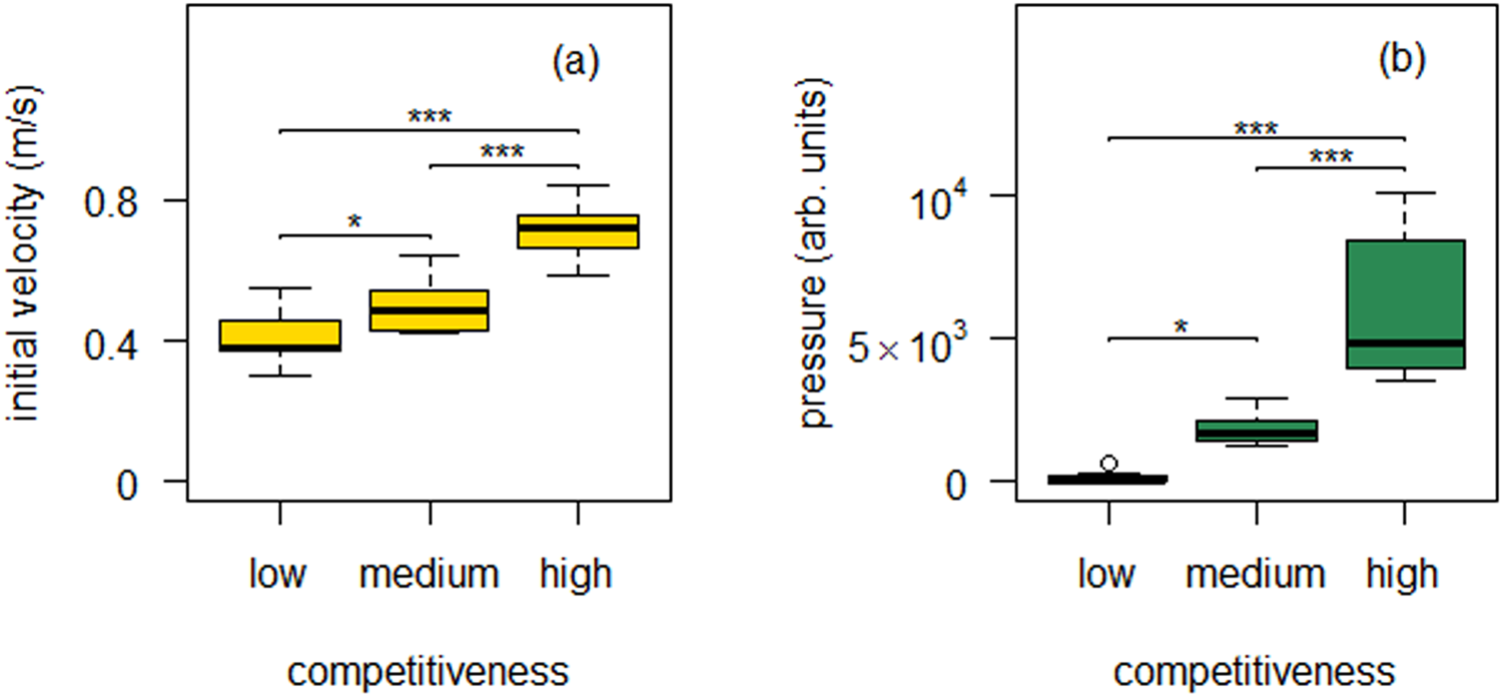
Quantitative characterization of competitiveness. (**a**) Initial group velocity which is used as a proxy for the desired velocity. The initial group velocity for each evacuation is calculated (see methods) and the boxplot corresponding to each competitiveness level (high, medium and low) is plotted. An ANOVA test (which rejected the null hypothesis of equal averages) was followed by a t-test with the Bonferroni correction for multiple comparisons. The p-values for the pairwise t-test are indicated as asterisks (*p-value < 0.05; ***p-value < 0.001). (**b**) Pressure at the doorjamb. The number of events during each evacuation run in which any sensor element was triggered on was monitored (see methods). Data were grouped as in (**a**) and a similar statistical test was performed.

Before dealing with the variables related to pedestrian movement, it is necessary to guarantee that the three series of evacuation drills are indeed performed at different competitiveness levels. In order to attempt a quantification of this we have focused on two magnitudes that are traditionally related to pedestrian competitiveness: i) the pedestrian desired speed and ii) the pressure that emerges due to the presence of persistent contacts. The pedestrian desired speed is characterized by measuring the initial group velocity at the very early stages of the evacuation, before people crowds in front of the door (see methods). The pressure developed among the individuals is a variable that, at this moment, is not accessible experimentally. As an alternative, in a previous work^20^ we have resorted to register the pressure developed at the doorjamb which, to our knowledge, is the first experimental attempt to quantify this crucial variable in pedestrian dynamics. As shown in Fig. 1 the correlation of both magnitudes (initial group velocity and pressure at the doorjamb) nicely correlates with the intended competitiveness degree. Interestingly, the statistical significance of the difference between the different cases also seems to be similar for both magnitudes: the p-value when comparing the highest and medium competitiveness is p < 0.001; whereas for the medium and lower competitiveness is p < 0.05. In summary, from this graph we can conclude that the level of competitiveness in the different sets of evacuations was clearly different as evidenced by the variation of the initial group velocity and pressure at the doorjamb.

**Figure 2:**
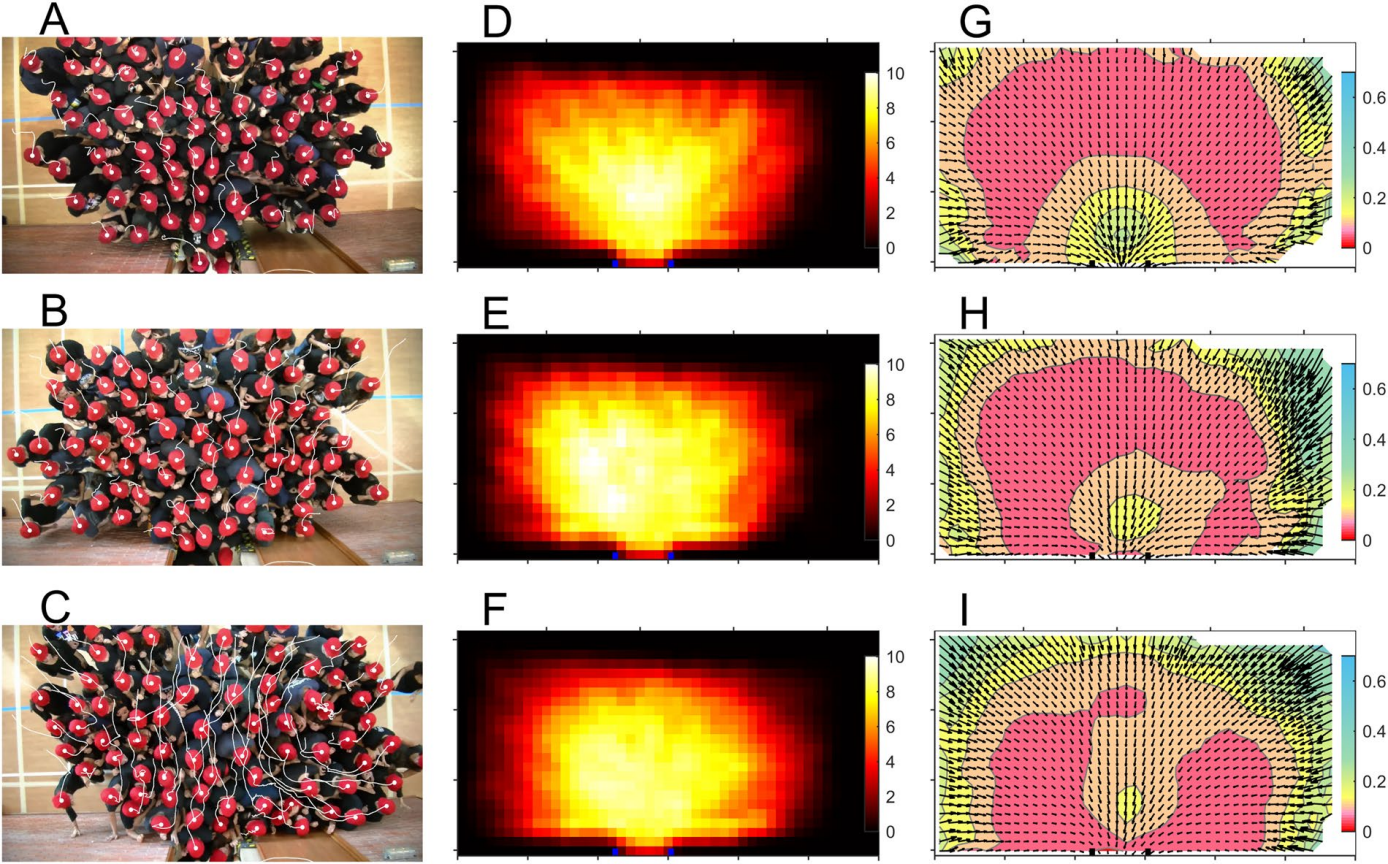
Left column (**A**–**C**): snapshots of the evacuation drill videos. With an image processing software the red hats have been marked with a white spot. Furthermore a white trail line is drawn linking the pedestrian positions during 2 seconds, so that longer trails correspond to larger velocities. Top (**A**), middle (**B**) and bottom (**C**) panels correspond to low, middle and high competitiveness (the same goes for the other two columns). Note that all the panels in the same column share the same scales. Middle column (**D**–**F**): Average densities obtained in the lapse time that goes from N = 10 to N = 50 (N is the number of persons that have left the room) for all the evacuation drills in a given competitiveness condition. In this case, the densities have been obtained with ω = 1. The colour scale at the left indicates the local density in persons/m^2^. Right column (**G**–**I**): average velocity fields obtained from N = 10 to N = 50) for all the evacuation drills in a given competitiveness condition. Arrows are the average velocities obtained with ω = 2 and the colour map is the average modulus of the velocity, |**v**|, with values as indicated in the colour bars.

Once we have characterized the competitiveness level in each of the three series of drills implemented, let us focus on the dynamics of pedestrian in their way out of the room. In the left column of Fig. 2 we show snapshots of the trajectories obtained in evacuations performed with low (Fig. 2A), medium (Fig. 2B) and high (Fig. 2C) competitiveness. Clearly, competitiveness triggers sudden collective movements in directions departing from the bearing towards the exit (see video 1 in Supplementary Material). Accordingly, the density maps (Fig. 2D–F) reveal a notable spatial dependence on the competitiveness. For the less competitive scenario (Fig. 2D) we found a V-shape of the isodensity curves, while for the more competitive evacuations the profiles become almost semi-circular. This suggests that, when the competitiveness is low, pedestrians do not tend to overtake each other, keeping their relative positions, hence leading to the observed V-shape. In competitive situations, however, pedestrians try to minimize their distance to the exit giving rise to the semicircular shape of the isodensity curves^24^. The velocity fields (Fig. 2G–I) also evidence the crucial role of competitiveness in the dynamics of the evacuation. Clearly, for the less competitive drills (Fig. 2G), the isovelocity curves are roughly circular, suggesting that the velocity mainly depends on the distance to the door: the closer to the door the higher the velocity. As competitiveness increases, it is observed that the regions to the sides of the door become slower whereas the one in front of the door becomes faster. This behaviour can be explained as follows: when pressure is high, the forces exerted by people at the sides compensate each other, preventing the movement. If, for some reason, these forces become unbalanced, a sudden movement towards one side occurs and pedestrians in the opposite side and in front of the door advance towards the exit. Furthermore, it is observed that competitiveness remarkably reduces the average velocity at the very door, a feature reminiscent of clogging^25–27^.

**Figure 3:**
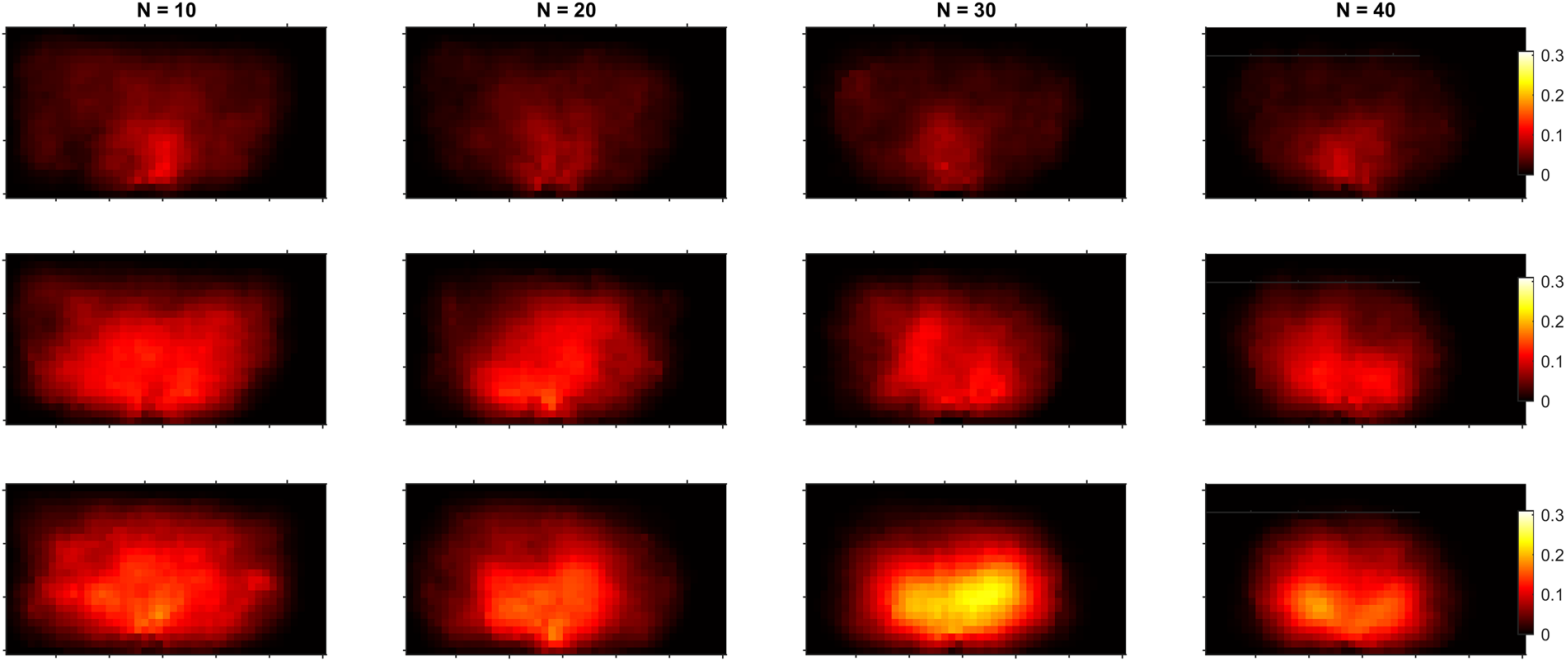
Temporal evolution of the fields of the kinetic stress like variable σ^k^. The three rows correspond to low, medium and high competitiveness (from top to bottom). The columns correspond to four different moments as labelled at the top, when N = 10, 20, 30 and 40 persons have left the room (the snapshots correspond to the kinetic stress averaged over the time it takes the next ten persons to leave the room, starting from N as indicated in each graph). The values of σ^k^ are shown by a colour scale which is shown at the right; note that all scales are the same. The axes tick spacing is 1 m.

From the velocity and density fields, we can straightforwardly obtain a magnitude σ^k^ (see methods) similar to the trace of the kinetic stress tensor. In pedestrian dynamics, this magnitude has been called “pedestrian pressure” and has been suggested to be correlated with the development of crowd turbulence^2, 14–17^. In our experiments, we have detected a strong temporal dependence of the obtained values. This is not surprising because as the evacuation draws on, the number of pedestrians in the room drops, and consequently the pressure decreases. In Fig. 3 we report the maps of σ^k^ obtained from the 10^th^ to the 50^th^ evacuated pedestrian, at intervals of 10 individuals (see methods). In the top row, for the low competitiveness scenario, σ^k^ decreases with time in a continuous and homogeneous manner. Similar behaviour is obtained for intermediate competitiveness although σ^k^ is considerably larger than in the previous case. For the high competitiveness scenario, the obtained values of σ^k^ are still larger. More noteworthy, a qualitative difference is observed as two high “pressure” lobes develop towards the sides of the room. These agree with the explanation given for the velocity map represented in Fig. 2I. Remarkably, for all competitiveness, the crowd pressure values are substantially above the 0.02 s^−2^ figure suggested to be the critical value at which turbulence develops^2^. This should be understood in the light of the different scenarios that are being compared: (a) a 44m-wide bridge where the behaviour of 1000–2000 people is analysed far from boundaries^2^; and (b) a narrow bottleneck. As expected, σ^k^ in a bottleneck is much higher than in a wide bridge.

**Figure 4:**
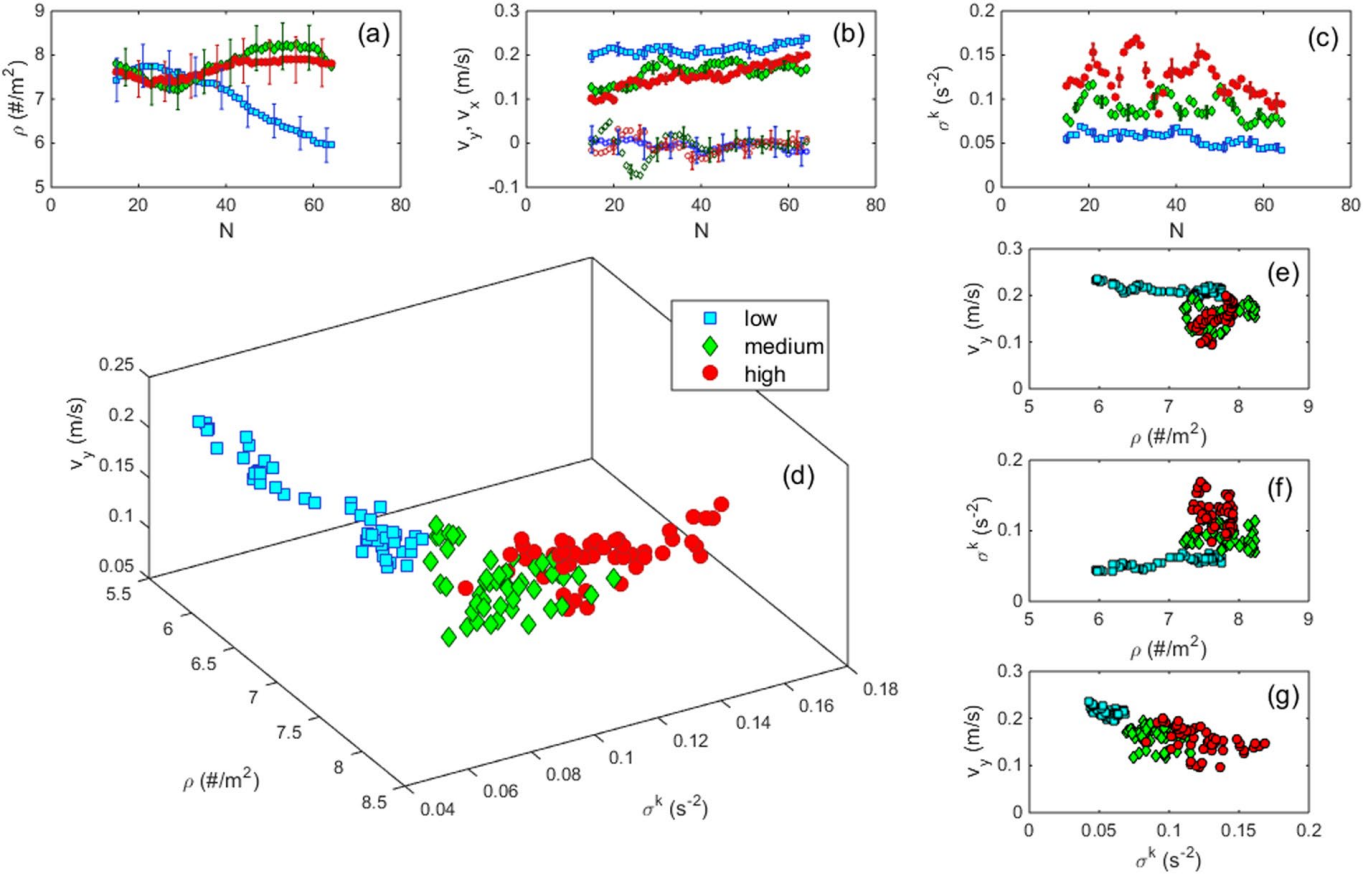
The average values of relevant variables in a 80 × 80 cm square in front of the door (located at a distance of 13 cm from the doorjamb to the closest side of the square) for different competitiveness as indicated in the legend. (**a**) The average density as a function of N. (**b**) v_y_ (solid symbols) and v_x_ (open symbols) as a function of N. (**c**) σ^k^ as a function of N. In a-c, the error bars indicate the 95% confidence interval of the values obtained, and are shown in only some data for each experimental condition in order to avoid overloading figures. (**d**) Three dimensional plot displaying the relationship between ρ, v_y_ and σ^k^. Plots (**e**), (**f**) and (**g**) are two dimensional projections of this graph.

In Fig. 4a–c we represent the temporal evolution of density (ρ), velocity (v) and the kinetic stress like variable (σ^k^) for a region near the outlet (a 80 × 80 cm square in front of the door). At this place, the evacuations start with very high values of density (close to 8 persons/m^2^) that do not seem to depend on the competitiveness (Fig. 4a). These figures remain roughly constant over time for the high and intermediate competitiveness scenarios, whereas they drop after N ≈ 35 for the low competitiveness case. The velocity values for the two components of the velocity (horizontal, v_x_, and vertical, v_y_, i. e. perpendicular to the exit) are displayed in Fig. 4b. As expected, v_x_ fluctuates around zero and fluctuations are more important for the medium and high competiveness scenarios than for the low one. A more distinctive behaviour is observed for v_y_: as predicted by the FIS effect, a higher velocity is reached for the low competitiveness case, which moreover seems to slightly increase over time as the density declines, in agreement with the known fundamental diagram^3^. The intermediate and high competitive cases reveal very similar trends: the velocity slightly increases over the whole evacuation. Surprisingly, this behaviour does not seem to correlate with the density values plotted in Fig. 4a, as could be expected from the fundamental diagram. Finally, as shown in Fig. 4c, σ^k^ exhibits three remarkable properties: 1) it correlates with the competitiveness degree: the higher the competitiveness, the higher σ^k^; 2) in all the scenarios, σ^k^ slightly reduces as the number of people in the room drops; 3) the temporal fluctuations of σ^k^ become more noticeable as the competitiveness increases. Importantly, the trends observed for the temporal evolution of these three variables are robust against changes in the way in which they are calculated, as shown in the methods section.

Now, starting from the results displayed in Fig. 4a–c, we investigate whether there is any correlation among the three different variables studied by representing a 3D plot (Fig. 4d) where the data seem to collapse into a single curve. To facilitate the understanding of this plot, we represent its two dimensional projections in Fig. 4e–g. The v_y_ vs. density plot (Fig. 4e) shows that for densities above 7.5 persons/m^2^, v_y_ can take values spanning from 0.1 to 0.2 m/s. This suggests that, for extremely dense situations, the velocity is not uniquely determined by the density as was believed. In the same line of reasoning, Fig. 4f shows that, for extremely dense situations, very different values of σ^k^ can develop for the same density values. Nonetheless, the data obtained from the different degrees of competitiveness seem to group around different values of σ^k^, in agreement with results of Fig. 4c. Finally, the graph of v_y_ vs. σ^k^ (Fig. 4g) reveals that the higher σ^k^, the smaller the velocity towards the exit, a behaviour that must surely be related to clogging^25–27^.

The results displayed in Fig. 4 and the statistical tests performed to analyse the differences among the different average quantities (see methods), suggest the necessity of more than one variable to characterize pedestrian dynamics. Traditionally, density has been thought of as the only parameter determining the collective behaviour, as reflected by the existence of a “velocity vs. density” fundamental diagram. This approach is correct for non-competitive pedestrians. If there is competitiveness, different behaviours can be obtained for the same (high) density values, hence advocating for the need of another variable. In principle, our results suggest that σ^k^ is a good one, as it correlates with the competitiveness degree and displays a relationship with the velocity in those situations where the density is not the control parameter any more. At this time, we cannot assert whether this increase of σ^k^ is the cause of velocity reduction, or just another side effect of competitive behaviour. It is likely that contact stress could also be a good candidate for the additional variable that completes the description. In fact, we know by indirect measurements at the door that contact pressure increases with competitiveness^20^. Interestingly, even in simpler systems formed by many inert bodies like granular materials, it has been already proved that completely different states can be achieved for ensembles with the same density, and by taking into account the contact stress it is possible to tell them apart^28–34^.

**Figure 5:**
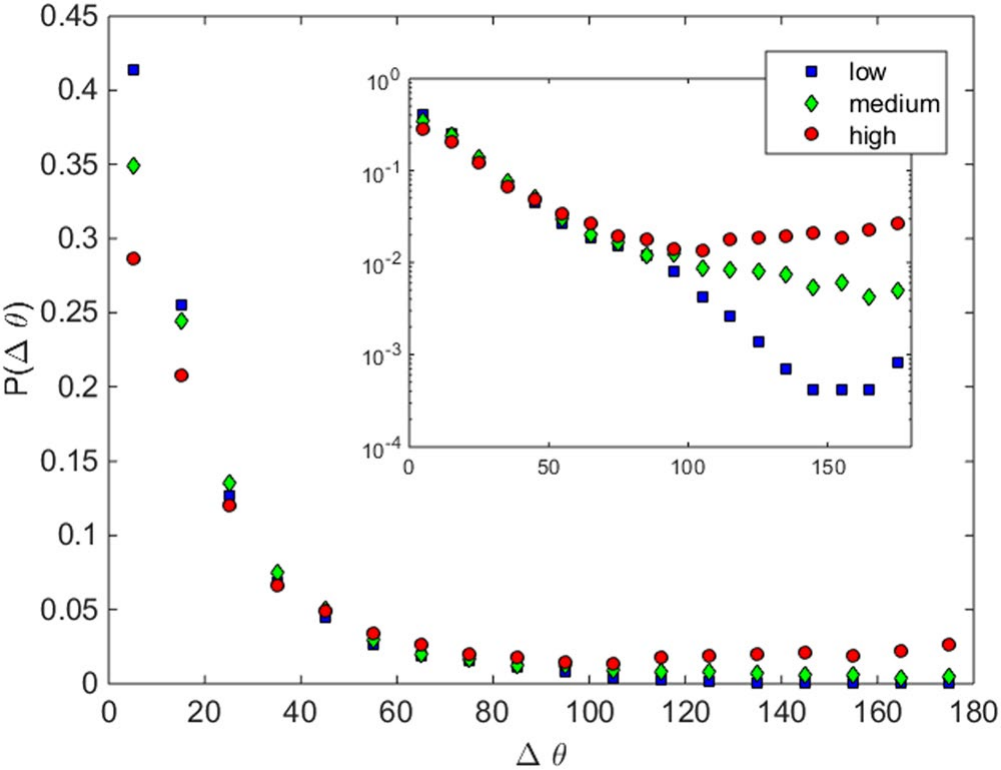
Normalized histogram of the velocity orientations with respect to that of the average velocity field. These have been calculated by obtaining the absolute deviation Δθ of the velocity field at each evacuation with respect to the average velocity field for all the evacuations in the class, in the interval N = 31 to N = 40 (a similar picture emerges for other intervals). Different symbols are used for each competitiveness degree as indicated in the legend. The inset shows the same data in semilogarithmic scale.

Finally, in order to highlight the appearance of undesired collective movements when the competitiveness increases, we have calculated the angular deviation Δθ of the pedestrian displacements with respect to the desired direction of movement. The latter is approximated by the average displacement on the basis of the fields represented in Fig. 2(g–i). The distribution of such deviations for the three competitiveness levels is enlightening (Fig. 5). First, in the main plot it becomes clear that reducing competitiveness enhances the amount of small values of Δθ; i.e. the number of displacements in the desired direction augments. Also, in the inset (in semilogarithmic scale) it is evidenced that increasing competitiveness leads to an important augment of large Δθ. Remarkably, the number of people walking backwards (i. e. in the opposite direction of the mean velocity field, or, Δθ > 90°) for the medium and higher competitiveness cases is rather large.

In summary, by means of analysing the trajectories of pedestrians evacuating a room under different conditions of competitiveness we have obtained new features of the flow that are attributed to the development of very high pressures within the crowd due to pushing behaviour. In particular, we find that the system properties are not determined by the density alone, evidencing the need to include (at least) one extra variable in the description framework. This finding will undoubtedly change the way in which pedestrian crowds in dense and competitive situations are studied, and will give rise to the development of new numerical methods that take into account more realistic contacts among the individuals.

## Methods

A series of evacuation drills were performed at the indoor gym of the University of Navarra with 95 volunteers aged about 22 years old. A detailed description of the evacuation procedures can be found in^19–21^. All the persons performing these exercises gave informed consent concerning their participation in the drills. They gathered for a whole morning and carried out three sets of evacuations: they were instructed to leave the room through a door which is 69 cm. wide, under three different set of rules. In the first set, 10 evacuations were done without intentional physical contact; in the second, soft physical contact was allowed (10 evacuations); and in the third, moderate pushing was allowed (13 evacuations). Those three sets of rules are referred to as low, medium and high competitiveness. The participants were instructed to enter a rectangular zone of 6 × 4 m (the door is at the center of the long side) so that their initial density was about 4 persons per square meter. Care was taken to avoid the formation of groups due to personal affinities; one of the organizers mixed the participants before each evacuation.

In order to characterize competitiveness by means of a quantitative variable, we have implemented two different alternatives. First, we measured the initial group velocity with which the pedestrians move towards the door at the beginning of the evacuation. This is expected to correlate with the desired velocity of the pedestrians as, during these early instants of the evacuation (before pedestrians crowd in front of the door), the physical interaction among the individuals is almost inexistent. The initial group velocity is calculated in the following way. Before the exit of the first individual, every consecutive pair of frames are compared, and the mean velocity is obtained. The initial velocity is the maximum mean velocity of these frames.

Secondly, we have innovatively monitored the pressure at one of the doorjambs. To this end, we employed a dynamic pressure sensor (TekScan®) containing 128 sensing elements arranged in a line 19.5 mm wide and 812.8 mm high, sampled at 100 Hz. The sensor was placed at a height going from 83 to 164 cm from the floor as, from previous drills, we know that the parts of the body that are more prone to get in contact with the doorjamb are the shoulders and the hips. After protecting the sensors with foam, we adjusted the sensibility of the sensing elements so that they were triggered when the pressure was above 3 kg/cm^2^. We then computed the total number of times that any sensing element had been triggered during the whole evacuation run, using this measurement as the variable to quantify the level of pressure during each experiment. The sensor was damaged during the experiment 27; therefore, for the highest competitive case, we only obtained six pressure values.

A video camera was installed inside the gym, above the door, and a region of 6 × 3.2 metres was recorded at 50 frames per second with a spatial resolution of 2250 × 1200 pixels (which is also the discretization grid for the calculations below). The volunteers were instructed to wear dark clothes and a red hat was given to them, so their heads can be tracked with an *ad hoc* image processing software. The main source of inaccuracy comes from the fact that sometimes (and especially during the high competitiveness drills) the hats of two or more persons come into contact. Although the hats are properly identified, the location of the centres as calculated from the image can be biased because only part of the hat is visible or correctly labelled. Taken also into account the motion of the head relative to the body, a resolution of about 10 cm is attained for the positions. If the sequence of the positions along time is filtered, the precision is still higher.

**Figure 6:**
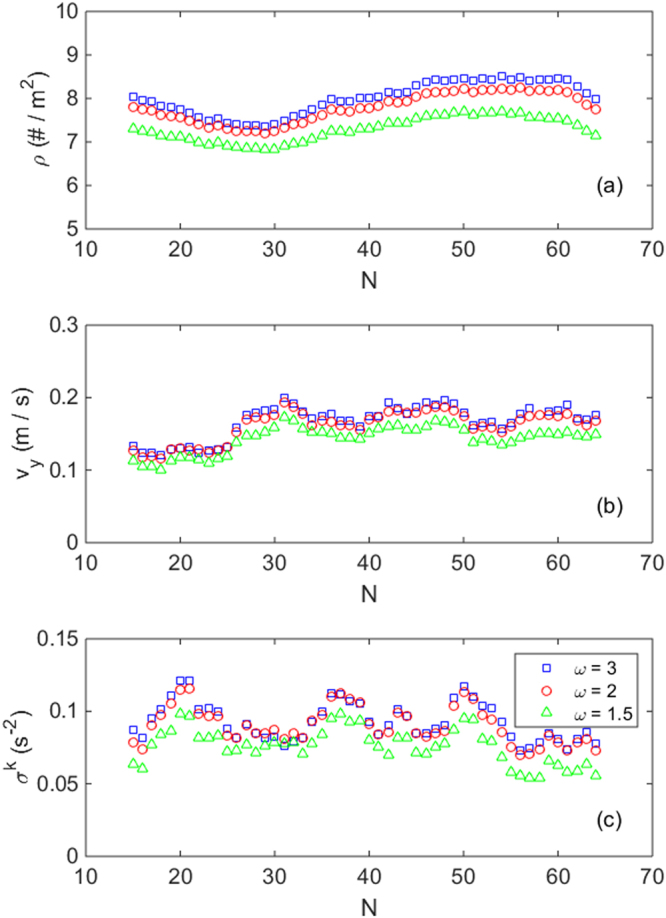
Influence of the coarse graining scale ω in the obtained values of (**a**) ρ, (**b**) v_y_ and (**c**) σ^k^ Vs N. As an example we report results for the intermediate competitiveness evacuations processed with ω = 1.5d, 2d and 3d as indicated in the legend. Note that ω = 2 is exactly the case shown in Fig. 4. It can be seen that the plots are all alike. A similar picture emerges for low and high competitiveness.

Once the tracks are obtained for each exit, we store them in a computer file containing labelled times and positions for individual persons. The instant velocity is calculated by measuring the distance travelled in a time interval of 1.5 seconds. This comparatively long time interval is used in order to smooth the oscillation produced by the gait. For every track, a value of the velocity is measured every 25 frames, which is the time (in order of magnitude) that a person changes its position by an amount of one diameter (for which we have taken d = 37 cm, the average shoulder to shoulder distance). The density and velocity fields are obtained by means of a coarse graining method^2, 23, 35, 36^. This method amounts to “spreading” the mass and the velocity of the object over a given area. In this way, these quantities which were formerly assigned to a point can provide a field. The mathematical procedure is just a convolution of the instantaneous locations with a coarse graining function $\varphi$, defining the spreading of the mass over the chosen area. In our case, $\varphi$ is a disk-shaped Heaviside theta with a diameter ω·d. We usually take ω = 2, which corresponds to twice the span between the shoulders of a person. The mass and the moment are then uniformly spread on this area with a normalizing condition (the mass and the moment must be conserved, and the sum of $\varphi$ must be one). Therefore we have for the density and the velocity fields the following expressions:

1 $$\rho ({\bf{r}},t)=\sum _{i}{m}_{i}\,\varphi ({\bf{r}}-{{\bf{r}}}_{i}(t))$$

2 $${\bf{V}}({\bf{r}},t)=\frac{{\sum }_{i}{m}_{i}\,{{\bf{v}}}_{i}\,\varphi ({\bf{r}}-{{\bf{r}}}_{i}(t))}{\rho ({\bf{r}},t)}$$

where *m*_i_ is the average mass of a person, **r**_i_ its position and **v**_i_ its velocity. In order to calculate mean fields, an average is performed in a square grid, the side of the square being 50 pixels of the image, equivalent to about 13 cm. The density and velocity fields are obtained in a straightforward way by taking the addition of all the contributions to each square after the coarse graining. Of course, the smoothing degree is dependent on the particular values chosen for the time intervals and lengths used to the discretization, as explained above, but we have checked that the velocity and density values do not change noticeably when choosing a different set of parameters. For instance, we tested a smaller or bigger coarse graining functions, changed by a factor of two (Fig. 6), another time interval for the calculation of the velocity, and several sizes of the grid square, from 25 to 75 pixels along the side, which corresponds to 6.7 and 20 cm respectively.

Once the velocity and density are obtained, a parameter akin the pressure can be calculated as the product of the density times the velocity variance: ${\sigma }^{k}({\bf{r}},t)=\rho ({\bf{r}},t)\cdot Var({\bf{v}})$. This has been termed the “kinetic stress” for a set of hard, discrete particles^21, 35^, and has already been applied to pedestrians^2, 12–14^. The variance of the velocity is calculated over time, i. e. $Var({\bf{v}})=\langle {[{\bf{V}}({\bf{r}},t)-{\bf{U}}({\bf{r}})]}^{2}\rangle$, where **U**(**r**) is the average velocity at time *t*. We have calculated the instantaneous density and the instantaneous variance of the velocity, and can therefore provide the instantaneous pressure. Let us remark that in our case the variance is performed by averaging over the different evacuation drills in the same conditions at the same moment. It does not make sense to calculate the average over time because as people leave the room the experimental situation changes (for instance, the number of people inside the room decreases with time). Moreover, instead of taking time as the independent variable, we argued^21^ that it is more sensible to consider the number of persons N that have already left the room. Irrespective of time, the same N will reference to the same condition, thus obviating the small time variability between drills. We have calculated the instantaneous values by averaging during the time it takes for 10 persons to cross the door, which is about 4 seconds, and the N value indicated in the axis of the graphs is the smallest ordinal in this window. The instantaneous variances of the velocity have been calculated by taking the differences of the velocity field at each drill ${\bf{V}}({\bf{r}},t)$ with respect to the mean velocity field ${\bf{U}}({\bf{r}})$ in all the drills during the time window defined by the exit of 10 persons. A map of this kinetic stress like variable (or pedestrian pressure) is thus obtained for every such window.

In order to establish quantitative comparisons among the different evacuation procedures, we have analysed the temporal evolution of different variables in a particular region close to the door: a 80 × 80 cm square, centred in front of the door, with the closest side at 13 cm from the doorjamb. In this square, we obtained the average velocity along the axis, i. e. towards the doorjamb (v_y_), the velocity along the perpendicular direction (v_x_), the kinetic stress, and the density. As before, the instantaneous values have been calculated by averaging in a window of 10 persons. In the plots some persons at the beginning and at the end of the evacuation are not represented in order to leave out the transients^21^.

We performed a statistical test to determine whether these average quantities display or not significant differences depending on the competitiveness degree. In particular, as the fluctuations are not Gaussian and the variances are widely different, we choose a Wilcoxon rank test (see Table 1 for the p-values; a permutation test was also performed and the resulting p-values are similar). For the density, the one corresponding to low competitiveness is significantly smaller than those corresponding to the intermediate and high competitiveness, which are not significantly different between them. The same goes for the velocity (it is higher for the low competitiveness, and not significantly different for the other two cases). Nevertheless, the kinetic stress is significantly different for each situation (all of them).

**Table 1:** P values resulting from Wilcoxon rank test when comparing the values of the density, velocity towards the exit (v_y_) and kinetic stress of the different competitiveness scenarios by pairs.

*competitivenessvariable*	low - medium	medium - high	low - high
density	p < 0.00001	p = 0.1	p < 0.00001
velocity v_y_	p < 0.00001	p = 0.06	p < 0.00001
kinetic stress	p < 0.00001	p < 0.00001	p < 0.00001

## Electronic supplementary material

Video Caption

Evacuation drill in a high competitiveness scenario
