# Supplementary material for: Pedestrian collective motion in competitive room evacuation
Source: Sci Rep. 2017 Sep 7;7:10792. doi: 10.1038/s41598-017-11197-x (PMC5589747; doi:10.1038/s41598-017-11197-x)
Supplement: Supplementary file 1 — Video Caption [file 41598_2017_11197_MOESM1_ESM.pdf]

Pedestrian collective motion in competitive room evacuation

A. Garcimartín[1], M. Pastor[1,2], C. Martín-Gómez[3], D. Parisi[4,5], I. Zuriguel[1].

Supplementary information.

Video Legend.

The video shows an evacuation drill for the case of high competitiveness. The videos are processed in order detect the positions of each pedestrian (marked with a white spot). Furthermore a white trail line is drawn linking the pedestrian positions during the last two seconds prior to the frame shown. Sudden collective movements appear in different directions, reminiscent of turbulent flow.
